# Sequence-Driven Classification of Aptamers by Target Type: Algorithm Development and Validation

**DOI:** 10.2196/92194

**Published:** 2026-11-13

**Authors:** Ava George, Andrew Takayama, Andres Medina, Amanda Reyes, Damian Ventura, Stevan Pecic, Ankita Mohapatra

**Affiliations:** 1 Department of Computer Science, Fullerton College, Fullerton, CA, United States; 2 Department of Chemistry and Biochemistry, California State University, Fullerton, CA, United States; 3 Department of Electrical and Computer Engineering, California State University, 500 N State Clg Blvd, Fullerton, CA, 92831, United States, +1 (657) 278-2853

**Keywords:** sequence-based classification, machine learning, systematic evolution of ligands by exponential enrichment, SELEX, DNA aptamers, decision trees, term frequency, inverse document frequency, extreme gradient boosting, XGBoost

## Abstract

**Background:**

DNA aptamers are short, single-stranded oligonucleotides that bind molecular targets with high affinity and specificity. Traditionally, aptamers are identified through SELEX (systematic evolution of ligands by exponential enrichment), an iterative process that is time-consuming and requires specialized equipment. To simplify and accelerate aptamer discovery, particularly across multiple molecular targets, computational approaches have gained momentum.

**Objective:**

In this study, we present a sequence-only framework for classifying DNA aptamers by target type using curated datasets of experimentally validated sequences. Target type here refers to the broad class of ligands that an aptamer binds to.

**Methods:**

Aptamer-target records were curated from the UTexas Aptamer Database, retaining only those sequences with complete annotations and a clearly defined target category. From these filtered records, the 2 target categories with the highest number of unique aptamer sequences—cell and steroid, comprising a total of 236 sequences—were selected for this study. Each sequence was then represented using character-level term frequency–inverse document frequency of n-gram motifs (n=3‐6) combined with guanine-cytosine content and sequence length. The dataset was then split into 70% training, 20% testing, and 10% validation sets. An ensemble of 9 bagged extreme gradient boosting classifiers was selected for its established performance on high-dimensional tabular data and native handling of class imbalance. The model was trained to distinguish between aptamers targeting cellular and steroidal molecules, with class weighting, oversampling, probability calibration, and per-class threshold optimization applied to address class imbalance. Model performance was evaluated using accuracy, macro-*F*_1_ scores, and recall on the test dataset and an independently generated external validation set comprising 13 new laboratory-generated steroid (aldosterone)–binding aptamers. The novelty of these laboratory aptamers relative to the training set was also confirmed using the pairwise Levenshtein distance.

**Results:**

The ensemble classifier achieved 97.92% accuracy and a macro-*F*_1_-score of 0.95 on a test set of 236 curated DNA aptamer sequences, demonstrating strong performance in distinguishing cell-binding from steroid-binding aptamers. The calibrated and threshold-optimized model was further validated on a set of 13 new laboratory-created aptamers for steroid aldosterone using experimental SELEX, achieving 100% recall and confirming its ability to generalize to new experimental data.

**Conclusions:**

These results demonstrate that informative, target-specific patterns can be directly extracted from DNA sequences, establishing a scalable, data-driven complement to SELEX in this preliminary proof-of-concept study for rapid prescreening of candidate aptamers. More broadly, this sequence-only approach could support faster, lower-cost triage of candidate aptamers, reducing reliance on iterative wet-laboratory SELEX as aptamer databases and target categories continue to expand.

## Introduction

### Aptamers and Their Applications

Aptamers are short, single-stranded oligonucleotides (DNA or RNA) that fold into 3D structures, enabling them to bind to molecular targets with high specificity and affinity. Compared with traditional antibody-based sensors, DNA aptamers in particular offer unique advantages. Aptamers used in vitro are chemically prepared oligonucleotides, and no animals or in vivo models are involved in their isolation, synthesis, or later identification. This makes the purification and reproducibility of aptamers much more efficient than the generation of antibodies. Next, because they are synthetically obtained, the potential for increased diversity in the oligonucleotide library pool can be achieved through simple chemical modifications using standard principles of nucleic acid chemistry. Finally, oligonucleotides are more stable than antibodies, making them ideal candidates for many biomedical applications [1]. These characteristics make DNA aptamers suitable for designing biosensors targeting various targets, especially for point-of-care diagnostics [2]. The ligands of aptamers may include but are not limited to ions, peptides, proteins, microbes (viruses and bacteria), and even entire cells [3,4]. In Figure 1, a 15-mer single-stranded DNA aptamer that folds into a 2-G-quartet structure and binds thrombin is shown. This interaction prevents thrombin from binding to fibrinogen and the subsequent coagulation cascade, thereby preventing blood coagulation [5]. The increase in nucleotide count in the aptamer allows greater variability in molecular structure, ultimately enabling it to adopt a unique fold [4,6].

**Figure 1.**
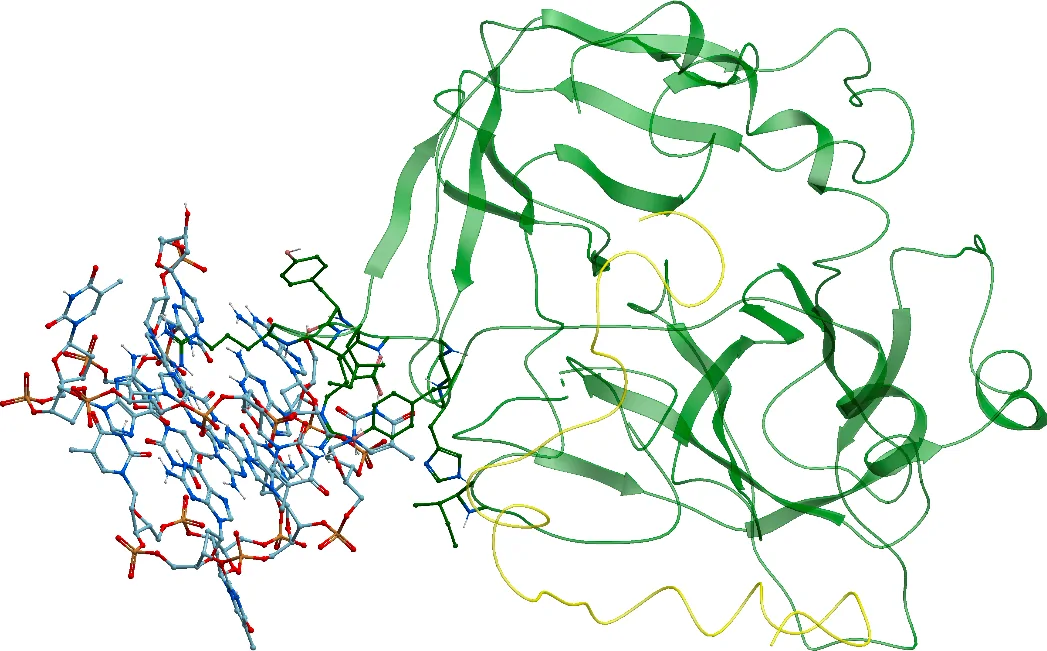
A crystal structure by Padmanabhan et al [7] (PDB: 1HUT) showing alpha-thrombin (in green) inhibited by a 15-mer single-stranded DNA aptamer, shown in blue and red. Figure prepared with ICM Pro (Molsoft LLC) [8].

An aptamer’s linear nucleotide sequence, also called the primary structure, determines its ability to fold into secondary and tertiary structures (Figure 2). This is based on the ability of nucleotide bases to interact with each other through Watson-Crick base pairing. These higher-order structures create unique 3D pockets that dictate target recognition sites and target specificity. Changes in these primary sequences can significantly alter folding patterns, which in turn change target selectivity and affinity to a specific molecular target. Hydrogen bonding, electrostatic interactions, π-π stacking, and van der Waals interactions contribute to the molecular recognition of a target as well as to an aptamer’s structural stability [8,9].

**Figure 2.**
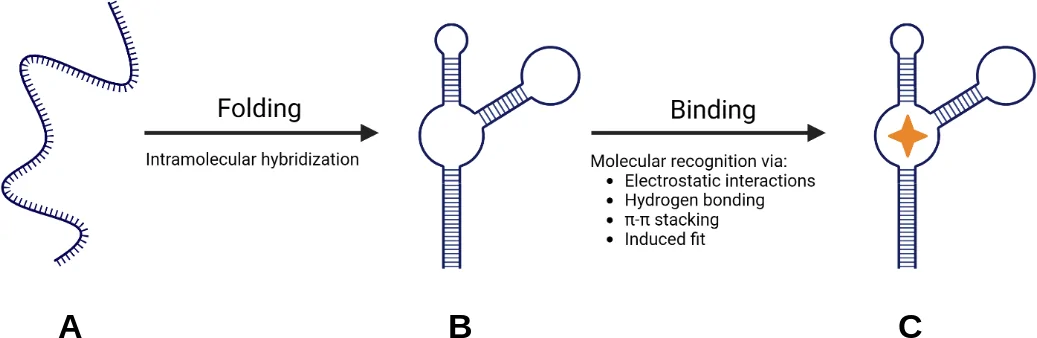
The primary (1°) structure of (A) a single-stranded DNA aptamer folding in on itself via intramolecular hybridization to form (B) its secondary 3D (2°) structure. (C) The 2° structure then has molecular recognition and undergoes a conformational change around its target (orange star). Figure prepared with BioRender (Science Suite Inc).

### Limitations of Traditional Discovery Methods

The traditional method of aptamer discovery, SELEX (systematic evolution of ligands by exponential enrichment), consists of several iterative rounds of binding and washing to select aptamers from a broad aptamer library, identifying high-affinity sequences for a specific target. Using the SELEX technique, aptamers have been selected for the amino acid tryptophan [10], the antibiotic chloramphenicol [11], the natural product theophylline [12], the neurotransmitter dopamine [13], and many other small-molecule targets. However, this process is time-consuming, expensive, and requires specialized equipment, and there are still many small molecules of interest for which isolating high-affinity aptamers remains difficult [10]. Under typical SELEX conditions, a target of interest is exposed to a library pool of single-stranded DNA oligonucleotides (20‐80 nucleotides in length), and sequences with affinity to bind the target noncovalently will be collected. In contrast, the nonbinding oligonucleotides will be eluted. Multiple cycles of polymerase chain reaction–based selection and amplification will promote competition among active compounds. They should eventually result in the purification of the few molecular species with the highest affinity or efficacy for a given target (Figure 3).

**Figure 3.**
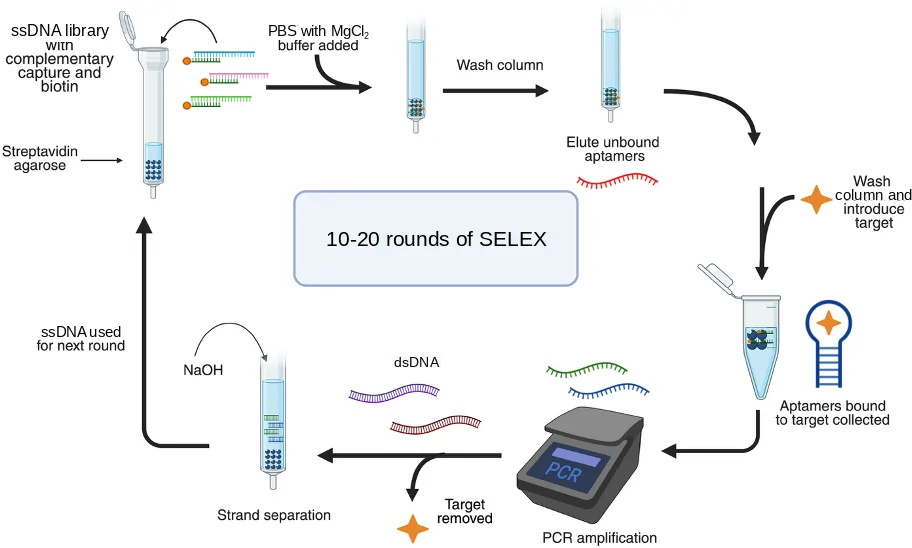
Schematic representation of the SELEX (systematic evolution of ligands by exponential enrichment) capture method. The starting point of the SELEX process requires a large library consisting of many randomized single-stranded DNA (ssDNA) sequences (eg, a nucleic acid library that has 30 randomized positions potentially contains 430 or about 1018 different oligonucleotide sequences) from which an aptamer will be isolated from. To capture SELEX, ssDNA aptamers are immobilized onto the column and target is then added. DNA aptamers bound to target are collected and amplified through the polymerase chain reaction (PCR). SELEX is composed of several rounds to produce a new pool of enriched ssDNA library. dsDNA: double-stranded DNA; PBS: phosphate-buffered saline.

Although methods such as high-throughput SELEX [14] and particle display [15] have significantly increased SELEX throughput, they are still optimized for single targets and can be resource-intensive. Therefore, there is a need to identify additional methods to reduce the complexity of SELEX by prior identification of aptamer candidates for the target of interest.

### Computational and AI-Driven Approaches

In the past few years, computational strategies have become popular to facilitate faster aptamer discovery and reduce the burden of traditional SELEX. The early *in silico* techniques for aptamer discovery mostly relied on structure-based modeling. For example, tools such as MFold (RNA Institute at the University of Albany) and NUPACK (Nucleic Acid Package; California Institute of Technology [Caltech]) use thermodynamic free energy minimization algorithms. All possible folding configurations of a single nucleic acid strand are evaluated, and the most thermodynamically stable structure is identified. Figure 4 shows examples of predicted secondary structures for aldosterone (ALDO) aptamer 14 using MFold (left) and NUPACK (right). These secondary-structure predictions can be used as inputs for tertiary-structure modeling on platforms such as RNAComposer or 3DRNA.

**Figure 4.**
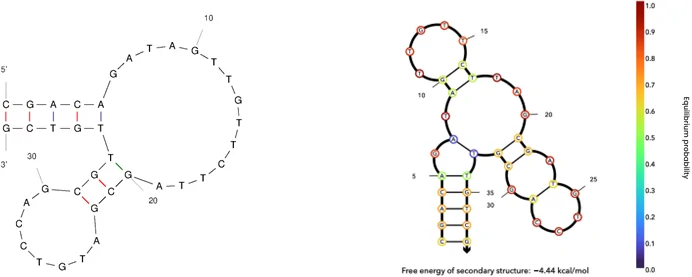
Predicted secondary structures of aldosterone aptamer 14. Generated images from MFold (left) and Nucleic Acid Package (NUPACK; right).

While these methods provide valuable insights into aptamer-target interactions, they are computationally expensive and difficult to scale to large aptamer libraries. They can fail for new targets, limiting their practical applications. Therefore, other data-driven methods were introduced, such as combining machine learning (ML) with genetic algorithms for finding novel RNA aptamers [11]. Other deep-learning methods can bypass explicit structural modeling and learn binding patterns directly from the primary sequence data. For example, AptaBLE (Aptamer Binding Language) uses pretrained encoders for aptamer and protein-pair inputs. The pairs are transformed into vectors representing the aptamer-molecule interactions, which in turn are used to predict binding strength or even generate new aptamer candidates [12]. However, these methods are target-specific and require retraining for each new target. This gap inspired the current work and is discussed in more detail in the following section.

### Identified Research Gap

Despite significant advancements from thermodynamics-based folding prediction tools (such as MFold and NUPACK) to deep-learning methods based on raw sequence data, many limitations remain in this area. First, much of the existing research, as well as available datasets, focus on RNA aptamers rather than DNA aptamers. DNA aptamers are highly preferred for biosensor fabrication because they offer greater chemical stability and lower synthesis costs [13]. Second, most ML models are target-specific and trained for a single molecule. For example, AptaBLE, discussed above, needs to be retrained each time a new target is introduced. Third, several of these models rely on accurate folding predictions, so an error at this critical step can propagate and amplify throughout the model. In summary, current research lacks a generalized, sequence-only framework for classifying DNA aptamers across multiple target categories based purely on primary sequence information. Addressing this gap enables rapid prescreening of aptamer candidates before experimental SELEX, ultimately reducing time and cost while supporting large-scale sensor design and discovery.

### Contributions of This Work

In this work, we present preliminary results of a sequence-based ML framework for classifying DNA aptamers by target type across diverse molecular classes. In this study, cells and steroids were selected as the targets of interest from the available UTexas database. Each aptamer sequence is represented using term frequency (TF)–inverse document frequency (IDF) rather than the raw sequence, and the resulting vector is then used as direct input to the classifier model. TF-IDF is a text-mining method that scores short sequence motifs (k-mers) according to how frequently they occur within an individual sequence (TF) relative to how commonly they occur across the entire dataset (IDF). Motifs that occur within one target class but are rare elsewhere are graded with a higher TF-IDF weight, tagging them as candidate class-discriminating features. TF-IDF was selected for this work because, unlike structure-based or deep-learning representations, it requires no folding prediction or a large target-specific pretraining set or per-target retraining, thereby directly addressing the limitations of prior approaches (discussed in the *Identified Research Gap* section).

This study aims to develop and evaluate a sequence-only framework for classifying DNA aptamers by target type without relying on structural prediction models and to assess whether the resulting classifier generalizes to independent, laboratory-generated sequences.

## Methods

### Dataset Curation and Filtering

For this study, we used curated entries from the UTexas Aptamer Database [16], which contains experimentally validated DNA aptamer sequences and their corresponding molecular targets. Only records with complete sequences and a clearly defined target category were retained. The categories with sparse representation (eg, proteins or peptides with few sequences) were excluded to reduce class imbalance and improve model robustness. After filtering, 2 dominant target classes were identified: cell-target and steroid-target aptamers. “Cell” entries in the dataset were defined as macroscale, whole-cell SELEX targets, and “Steroid” entries were classified as small, low-molecular-weight hydrophobic ligands. The resulting datasets contained unique single-stranded DNA aptamer sequences, each labeled according to its target class.

### ML Pipeline for Aptamer Classification

Each sequence was standardized by converting to uppercase, removing ambiguous characters, and trimming whitespace to ensure fair class representation and balanced training. We applied class-weighting and random oversampling of the minority class (steroid) before training. Finally, a separate, independent dataset of 13 newly discovered aptamer sequences from our laboratory was prepared in the same format for testing. These sequences, experimentally verified to bind to steroid targets, were used exclusively to assess the model’s generalization capability beyond the training database.

Each DNA aptamer sequence underwent a 2-step feature-engineering process to transform it into a format better suited as input to the model. To capture local sequence motifs that could influence binding affinity, each aptamer sequence was decomposed into overlapping subsequences ranging from 3 to 6 nucleotides in length (character-level n-grams, n=3‐6). For example, the sequence “ACGTACGT” will generate 6-mer motifs such as “ACGTAC” and “CGTACG,” 4-mer motifs such as “ACGT,” and “CGTA,” and 3-mers such as “ACG” and “CGT.” These variable-length subsequences were then vectorized using a TF-IDF [17]. TF measures how often a k-mer motif occurs in a single aptamer sequence (Equation 1).


(1) $$\begin{array}{l} TF(k,s)=\frac{\text{Number of times k-mer motif}\;k\;appears\;in\;a\;sequence\;s}{Total\;number\;of\;\text{k-mer}\;motifs\;in\;sequence\;s} \end{array}$$

IDF measures how common the same k-mer motif is across all sequences in the entire dataset (Equation 2). A low IDF is a sign of a k-mer being common across multiple target groups, and is therefore potentially irrelevant for specific binding to a target class. A high IDF value is therefore preferred.


(2) $$IDF(k)=\log\left(\frac{Total\;number\;of\;sequences\;in\;the\;dataset}{Number\;of\;sequences\;containing\;the\;\text{k-mer}\;motif\;k}\right)$$

The TF-IDF factor (Equation 3) was then calculated. This helps identify motifs that frequently occur in a target set but are rare across the entire dataset, suggesting that those motifs may contribute toward binding with the target.


(3) TF-IDF (k,s)=TF (k,s)×IDF (k)

In addition to the TF-IDF, 2 global sequence-level features were also computed: guanine-cytosine (GC) content and total sequence length, which were also standardized and concatenated with the TF-IDF vector to form the final feature matrix used to train the classifier. The vector, therefore, now contains both local motif-level and global sequence-level properties solely derived from the primary DNA sequence, without requiring any structural predictions or folding models. To understand which sequence properties most influenced classification, feature importance was also determined for each of the 9 bagged models using gain (the improvement in model loss attributable to a feature across all splits of a model). Importance values were averaged across the ensemble to identify k-mer motifs and descriptors globally relevant to classification.

### Model Selection

The classification model was built on an ensemble learning framework using extreme gradient boosting (XGBoost) decision trees. XGBoost was selected for this evaluation based on three primary reasons: (1) its performance is well established on structured, high-dimensional tabular data such as TF-IDF feature vectors; (2) it provides native support for handling class imbalance by assigning class weights; and (3) considering the modest size of the dataset, XGBoost has a lower chance of overfitting compared to more complex model architectures. As this study is intended primarily as a proof-of-concept for sequence-only target-type classification rather than comparative benchmarking of ML algorithms, a systematic comparison against alternative classifiers was considered outside the scope of this evaluation. Comparative model benchmarking is noted as a direction for future work (see the *Future Work* section).

A bagged 9-ensemble model strategy was used to reduce variance and improve generalization stability on a relatively sparse dataset. Each subdataset was split into 70% training, 20% testing, and 10% validation subsets. The validation subset was used for early stopping during training. Each model was trained on a unique bootstrapped subsample of the entire training set using a distinct random seed. The XGBoost classifier was trained with a multinomial softmax objective, using regularization parameters to prevent overfitting, including constraints on tree depth, minimum child weight, and subsampling rates. The number of boosting rounds was controlled by early stopping based on the fixed validation set (with multiclass log-loss).

### Data Analysis

Model performance was evaluated using accuracy, macroaveraged *F*_1_-score, and per-class precision/recall to account for the imbalance between cell- and steroid-binding aptamers. Confusion matrices were used to visualize class-level classification patterns. Following training, the output probabilities from all 9 ensemble models were averaged at the soft-probability level. These averaged probabilities were then subjected to a post hoc calibration step using a logistic regression model trained on the same validation set, thereby improving the interpretability and reliability of the predicted confidence values. Finally, we implemented a per-class threshold optimization to identify decision thresholds that maximize the macroaverage. The trained, calibrated ensemble was then used to generate predictions for the held-out test set. To quantify the stability of reported performance metrics, given the small test set size, 95% CIs for accuracy and macro-*F*_1_ were estimated via the nonparametric bootstrap resampling of test-set predictions for both the argmax and threshold-tuned decision rules.

In addition to the model’s performance on the curated dataset, we evaluated its generalization to an independent set of experimentally generated DNA aptamer sequences, which is discussed in detail in the *In Vitro Aptamer Selection and Validation* section. These sequences were discovered through laboratory screening using SELEX, targeting steroidal molecules, and were not part of the original aptamer database or any training/calibration subsets used during model development. The external dataset served as a true out-of-domain validation benchmark, representing the type of novel aptamers encountered during early-stage discovery. To ensure consistency, each sequence from the external set underwent the same preprocessing and feature transformation pipeline as the training data, including standardization and TF-IDF feature extraction, without retraining or refitting any part of the model. Moreover, to ensure that memorization of near-duplicate training sequences is not a factor, sequence novelty was computed for each of the 13 new sequences as the normalized Levenshtein distance to its nearest neighbor in the training set. Finally, to assess whether calibrated model confidence tracked binding strength, the Spearman rank correlation between each aptamer’s calibrated steroid-class probability and its measured *K*_d_ was computed. The graphical overview of the process is presented in Figures 5 and 6.

**Figure 5.**
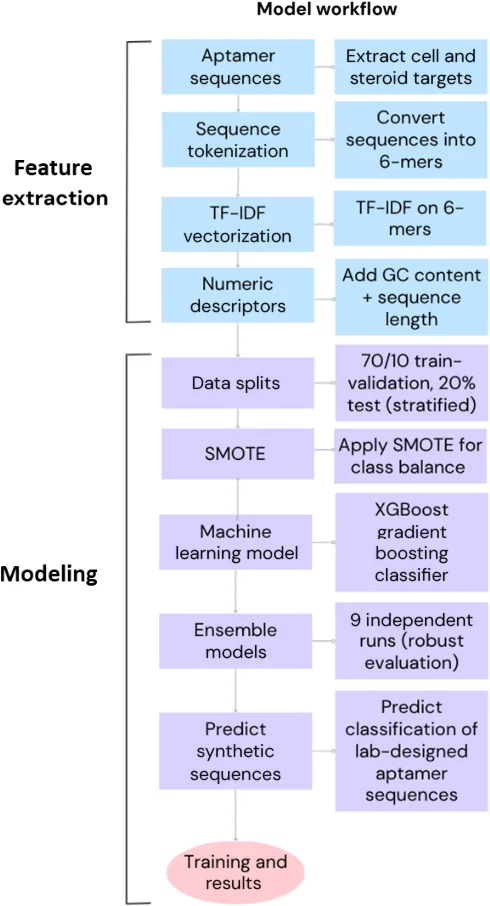
Data preprocessing and model design. GC: guanine-cytosine; SMOTE: synthetic minority oversampling technique; TF-IDF: term frequency–inverse document frequency; XGBoost: extreme gradient boosting.

**Figure 6.**
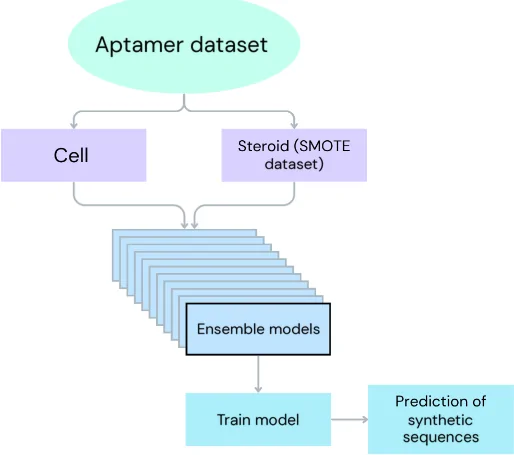
Ensemble model design. SMOTE: synthetic minority oversampling technique.

Full hyperparameter values and training configurations are available in the code repository, mentioned in the *Data Availability* section. Dataset preprocessing and model training were performed in Python. Secondary-structure analyses of the newly isolated aptamers in the laboratory were performed using MFold and NUPACK. Binding confirmation assays used Thioflavin T (ThT) fluorescence measurements, with curve fitting on GraphPad Prism 10 (described in detail in the *In Vitro Aptamer Selection and Validation* section).

### In Vitro Aptamer Selection and Validation

Unless otherwise stated, all reagents were used without further purification. All oligonucleotides and modified oligonucleotides (biotinylated, fluorescein, and dabcyl-tagged) were purchased and purified via reverse-phase high-performance liquid chromatography from Sigma Aldrich. ALDO was purchased from Sigma Aldrich. Buffers, 10× concentrated phosphate-buffered saline (PBS) and 10× concentrate tris-borate-ethylenediaminetetraacetic acid, were obtained from Corning. Streptavidin agarose resin was obtained from Thermo Scientific. Mini gravity columns were purchased from Biorad. Ultracentrifugal filter units (3K and 10K) were purchased from Millipore. DNase/RNase-free water was used for all purposes unless otherwise noted. All cloning materials were purchased from Fisher Scientific. For this selection, the oligonucleotides were obtained and used following our previously published library design shown in Textbox 1.

Textbox 1. **DNA library and primers used for SELEX (systematic evolution of ligands by exponential enrichment).**

1. 72-mer library with random N30 bases: 5′-GAGGCTCTCGGGACGAC(N30)GTCGTCCCGATGCTGCAATCGTAA-3′

2. Forward-primer (18-mer): 5′-GGAGGCTCTCGGGACGAC-3′

3. Reverse-primer (22-mer): 5′-TTACGATTGCAGCATCGGGACG-3′

4. Biotinylated reverse-primer (22-mer): 5′-biotin-TTACGATTGCAGCATCGGGACG-3′

5. Biotinylated column immobilizing capture (18-mer): 5′-GTCGTCCCGAGAGCC-ATABioTEG-3′

The SELEX reaction buffer used for this selection procedure was 1× PBS concentrate with 2 mM of MgCl2 and pH adjusted to 7.4. The ALDO stock solution was prepared at 10 mM in 200-proof ethanol and stored in a refrigerator. A fresh solution of ALDO at the desired concentration was prepared before each round of SELEX by dissolving the ALDO stock solution in the SELEX buffer. The 0.5× concentrate tris-borate-ethylenediaminetetraacetic acid buffer was used as an electrophoresis buffer. The strand separation buffer was 1× PBS concentrate without MgCl_2_. All oligonucleotides were dissolved separately in DNase/RNase-free water to prepare 100 μM stock solutions of each, which were divided into small aliquots sufficient for one SELEX round, and stored at −20 °C. The selection process has been described previously by us [14,15,18].

A 750 μL aliquot of 100 μM ThT solution was mixed with an equal volume of 10 μM aptamer solution and incubated for 40 minutes at room temperature, protected from light. Ten tubes were labeled, and 125 μL of SELEX buffer with 2 mM MgCl_2_ was added to tubes 2 to 10. In tube 1, 250 μL of 200 μM ALDO was added. Serial dilutions were made up to tube labeled 9. The tube labeled 10 did not contain ALDO. This was followed by adding 125 μL of ThT/aptamer solution to each tube. The mixtures were incubated for 40 minutes at 30 °C, protected from light. Measurements were conducted in triplicate in 384-well black plates (Costar) using a microplate reader M2 (Molecular Devices) to record ThT fluorescence spectra at excitation and emission wavelengths of 425 and 490 nm, respectively. The results were analyzed using GraphPad Prism 10 using nonlinear regression curve: curve fit, exponential, one phase decay [19].

## Results

### Performance of the ML Pipeline for Aptamer Classification

The ensemble classifier achieved strong performance on the held-out test set. Using calibrated probabilities with a standard argmax decision rule, the model obtained an accuracy of 95.83% (95% CI 89.58-100.00) and a macro-*F*_1_-score of 0.94 (95% CI 0.83-1.00; Figure 7). After applying class-specific threshold tuning, overall accuracy improved to 97.92% (95% CI 93.75-100.00) with a macro-*F*_1_ of 0.97 (95% CI 0.89-1.00), indicating that threshold optimization enhanced minority-class recovery without loss of precision. The confusion matrix (Figure 7) shows that the model consistently identified steroid-binding sequences while only misclassifying a small number of cellular-class aptamers. These results confirm that short-range and mid-range sequence motifs, combined with minimal global descriptors (GC content and sequence length), are sufficient to distinguish between aptamers targeting cellular and steroidal molecules.

**Figure 7.**
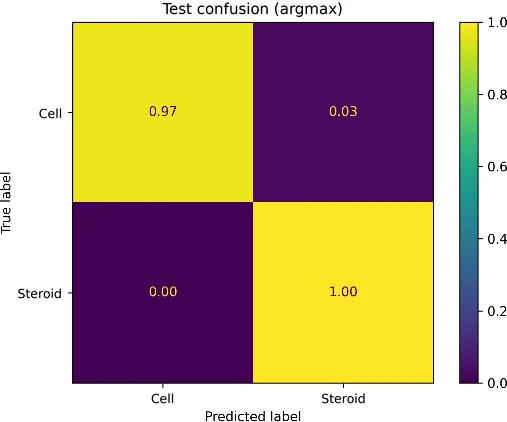
Confusion matrix for UTexas dataset performance.

### Feature Importance Analysis

The gain-based feature importances, averaged across all ensemble models, are shown in Figure 8. Several of the short-string motifs are substrings of the longer (6-mer) sequences, as expected, because overlapping character n-gram windows capture the same underlying patterns. In addition to motif-level contributions, sequence length was identified as a frequently used feature based on weight-based importance. Given the sequence length in the feature importance list and its weight-based ranking (Table 1), we examined whether sequence length differed between the 2 target classes. Cell-binding aptamers were significantly longer (n=187; mean 83.4, SD 25 nt) than steroid-binding aptamers (n=49; mean 63.1, SD 19.5 nt; Mann-Whitney *U*=6917.0, *P*=3.9 × 10⁻⁸; Figure 9). In this example, while this finding indicates that sequence length holds a genuine class-splitting signal, it should not be correlated to the size of the target in general, that is, the same-sized library can be used not only to detect small molecules successfully but also for selection of large molecules, such as proteins and enzymes, that is, their active sites. In most cases, the sequence length is between 20 and 80 base pairs, and there is no specific protocol defining which library to select for SELEX. In this study, we used a random (N30) library: 5′-GGAGGCTC­TCGGGACGAC (N30) GTCGTCC CGCCTTTAG­GATTTACAG-3′ [14].

**Figure 8.**
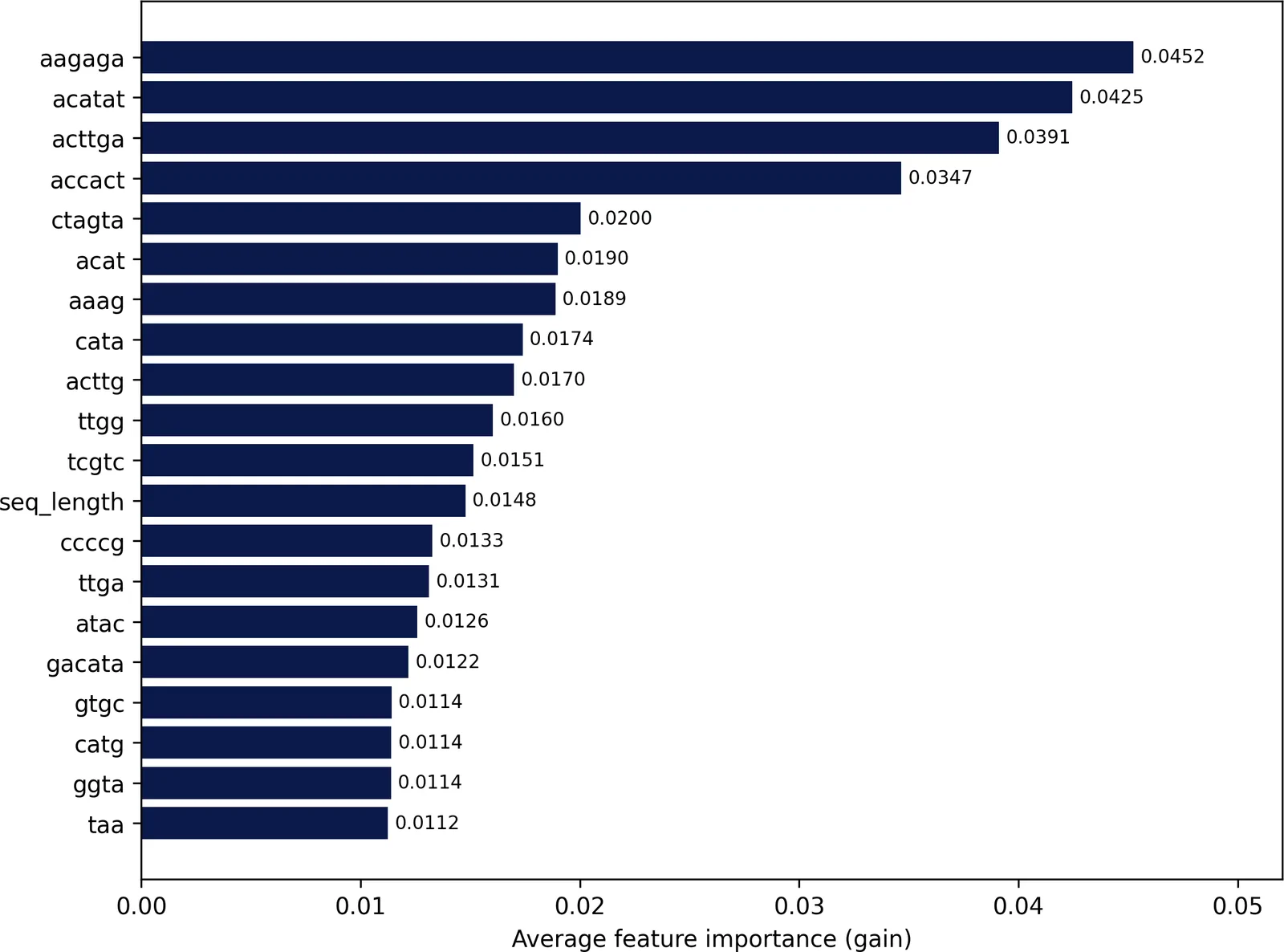
The best 20 features by average gain across all ensemble models.

**Table 1.** Weight-based feature importance.

Feature	Weight
seq_length	129
tag	59
aca	40
gta	39
gac	35
tac	32
cga	28
ttga	28
aag	25
tga	22
act	21
atg	21
ggta	21
tgg	21
cata	20
ccg	20
gagg	18
agtt	17
taa	17
tat	17

**Figure 9.**
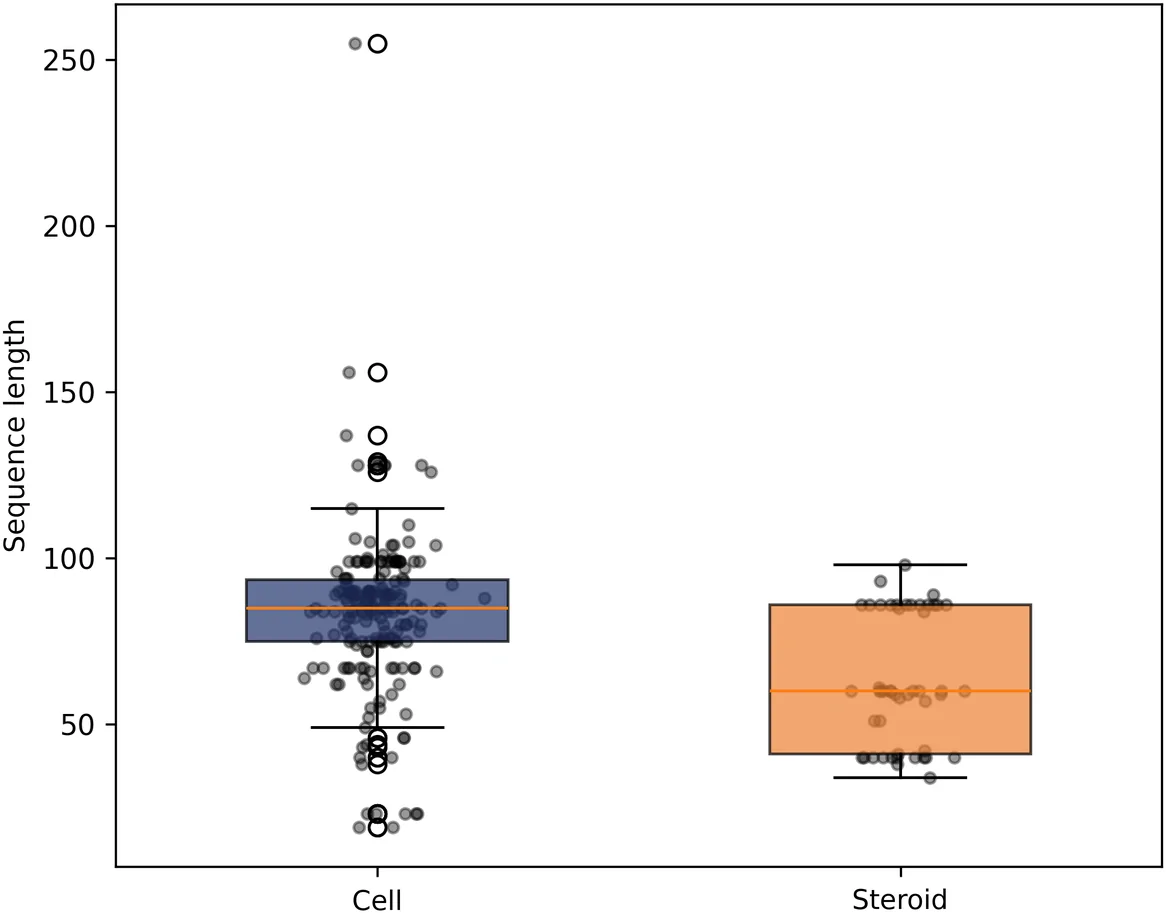
Sequence length by target group.

### Experimental Validation of In Vitro Selected Aptamers

The 13 aptamers isolated in our laboratory are shown in Table 2. All sequences in this set were experimentally confirmed to bind to ALDO. The ALDO aptamer set shows strong sequence similarity but different ThT affinities, implying that relatively small sequence changes shift the 3D folding and binding affinities of aptamers for ALDO. Most sequences share conserved flanking motifs typical of a selected library scaffold, that is, frequent ACGACG/CGACG located at the 5′ and GC-rich termini (eg, AGTCGT/TGTCG), while the internal regions vary in length and composition. This pattern suggests that the main molecular recognition motifs for ALDO binding are not the specific base composition but rather a specific secondary-structure arrangement (eg, an exposed stacked stem, internal bulge, or groove) that can accommodate this steroidal target.

**Table 2.** New aptamer sequences isolated in this work.

Target	Number of ALDO^a^	Sequence	ThT^b^ *K*_d_^c^ (μM)	MFold (kcal/mol)	NUPACK^d^ (kcal/mol)
Steroid	14	CGACAGATAGTTGTTCTTAGCGATGTCCAGCGTTGTCG	2.248	−3.28	−4.44
Steroid	15	ACGACGACAGTGCCTTGATATACGTTGGGCTGGTAGTCGT	26.2	−8.14	−8.26
Steroid	05	GACGACGAGACCCTTGATACTTCAAGCGTCAGCGAAGTCGTC	0.7306	−11.21	−10.94
Steroid	11	CTGGGGTGCAATTCTTATGTAACGGGTCCCGG	3,994,244	−2.99	−3.26
Steroid	17	CGACCGGAGTGTCTTAGTGTATAACTGAGTTTAGGTCG	29.57	−6.89	−5.59
Steroid	23	CGACGGTAGGTAGGCCAACTGGGTATTTACTGGTGTCG	281.8	−3.95	−2.53
Steroid	16	CGACAAATCTTTGGTGTGATGGATGTTGTCTTGTGTCG	2,632,540	−4.46	−4.83
Steroid	22	ACGACGACCGGTCCCTGTTGTTAGGAATAGGGGGAGTCGT	4,945,384	−9.18	−8.17
Steroid	06	ACGACGATGGGCCCCGATATGTACATTTCGGGTGAGTCGT	0.3785	−9.99	−10.20
Steroid	13	GGACGACGGAGACCCCATGTTTTCTGGTGTCAGCGTAGTCGTCC	225.7	−10.08	−10.13
Steroid	10	ACGACGGAGGGTCCCGTTGTTCTACGATGGGTTTAGTCGT	36.68	−8.56	−7.75
Steroid	02	CGACGGGACACTTTGTATGTAAAGTGAGGTTCACGTCG	107,369	−11.63	−11.52
Steroid	07	ACGACGGGTGAGGTTCTTTATAACTGATGGGCCCTGTCGT	3,332,006	−7.31	−7.54

a ALDO: aldosterone.

b ThT: Thioflavin T.

c All *K*_d_ graphs have been provided in Figures S1 to S25 in Multimedia Appendix 1.

d NUPACK: Nucleic Acid Package.

ThT K_d_ values range approximately 7 orders of magnitude, separating the selected aptamers into several subgroups. The tightest ALDO binders are ALDO 06 and ALDO 05 with K_d_ values of 0.3785 µM and 0.7306 µM, respectively, followed by ALDO 14 with a *K*_d_ value of 2.248 µM. Moderate-affinity ALDO binders include ALDO 15, ALDO 17, and ALDO 10, with *K*_d_ values in the ThT assay of 26.2 µM, 29.57 µM, and 36.68 µM, respectively. Weaker binders include ALDO 13 (K_d_=225.7 µM) and ALDO 23 (*K*_d_=281.8 µM). At the same time, several sequences show no binding under these conditions: ALDO 02, ALDO 11, ALDO 16, ALDO 22, and ALDO 07, with *K*_d_ values above 10,000 µM. We believe these nonbinding aptamers probably still bind to ALDO but likely fold into compact, thermodynamically stable complexes that are not suitable for ThT assay conditions. This is something that needs to be explored in the future—that is, determining *K*_d_ values for these sequences using other fluorescent assays and conditions. We tried to correlate the obtained in vitro *K*_d_ values to predicted folding free energies and observed that stability alone does not predict their binding to ALDO. Both MFold and NUPACK predict a wide range of stabilities, with MFold values ranging from −2.99 to −11.63 kcal/mol and NUPACK from −2.53 to −11.52 kcal/mol. The best aptamer binders, ALDO 06 and ALDO 05, are predicted to be fairly stable in both MFold and NUPACK, supporting the idea that a defined fold is necessary for high-affinity ALDO interactions. However, other examples demonstrate that high predicted stability is not sufficient, exemplified by ALDO 02, which is among the most stable by both models (MFold −11.63 kcal/mol and NUPACK −11.52 kcal/mol) but shows no binding to ALDO (>10,000 µM). On the other hand, ALDO 14 shows good binding affinity for ALDO with a *K*_d_ value of 2.248 µM, while relatively weak predicted stability (MFold −3.28 kcal/mol and NUPACK −4.44 kcal/mol), implying that the geometry of the fold (even if only modestly stable) can be more important than ΔG magnitude. Taken together, these comparisons indicate that ALDO *K*_d_ values are primarily sensitive to structural class/topology (which elements are exposed and how bases stack). In contrast, MFold/NUPACK ΔG values largely predict overall base-pairing stability without considering the formation of the target-binding complexes.

To evaluate generalization beyond the curated database, these aptamers were fed to the completely trained ensemble model and were not a part of the training or validation datasets. To assess the novelty of each of the 13 new laboratory-created aptamer sequences relative to the UTexas Aptamer Database sequences used to train the model, we calculated pairwise sequence similarity between the 2 groups using the normalized Levenshtein distance. The mean normalized distance between each of the 13 laboratory aptamers and its nearest neighbor in the training set was 0.40 (SD 0.07), with no sequence sharing more than 75% similarity with an aptamer in the training sequences. This confirmed that the 13 aptamers are genuinely unique, and the model’s performance on these sequences shows generalization rather than memorization. Detailed information on the Levenshtein distances for each of these sequences is provided in Table 3.

**Table 3.** Levenshtein distance results for the new aptamer sequences.

Aptamer number	Nearest training sequence	Raw test sequence	Normalized distance	% identity
14	CTCGGGACGACAGATAGTTGTTCTTAGCGATGTTCAGCGTTGTCGTCACCC	14	0.2745	72.55
15	ACGACGGTGGCATGAACATAAGTTCCCCCTTGAAAGTCGT	15	0.375	62.50
5	GACGACGCCCCATGTTCCATGGATAGTCTTGACTAGTCGTC	16	0.381	61.90
11	CTCGGGACGACAGATAGTTGTTCTTAGCGATGTTCAGCGTTGTCGTCACCC	25	0.4902	50.98
17	CGACTTAAGGTATGTGATCTTAGTTGTAGTTCAAGTCG	15	0.3947	60.53
23	CTCGGGACGACGGTAGGTAGGCCAACTGGGTATTTACTGGTGTCGTCACCC	13	0.2549	74.51
16	CGACTTAAGGTATGTGATCTTAGTTGTAGTTCAAGTCG	16	0.4211	57.89
22	ACGGGATGTCCGGGGTACGGTGGTTGCAGTTCGT	18	0.45	55.00
6	ACGGGATGTCCGGGGTACGGTGGTTGCAGTTCGT	17	0.425	57.50
13	GACGACGCCCCATGTTCCATGGATAGTCTTGACTAGTCGTC	15	0.3409	65.91
10	ACGGGATGTCCGGGGTACGGTGGTTGCAGTTCGT	15	0.375	62.50
2	CGACTTAAGGTATGTGATCTTAGTTGTAGTTCAAGTCG	15	0.3947	60.53
7	ACGGGATGTCCGGGGTACGGTGGTTGCAGTTCGT	19	0.475	52.50

While both raw and calibrated argmax misclassified a subset of borderline cases, the final calibrated and thresholded ensemble successfully identified all 13 sequences, achieving 100% recall. To explore if the model confidence scores reflect binding quality, we calculated the Spearman rank correlation between the calibrated steroid-class probability and experimentally measured *K*_d_ values for the 13 laboratory-generated aptamers. An insignificant negative correlation (ρ=−0.05, *P*=.08), indicating no statistically significant or practically meaningful relationship between calibrated steroid-class probability and experimental *K*_d_ values, suggests that the model assigned higher confidence to sequences with lower *K*_d_ values (ie, better binding). Although this test did not reach statistical significance at the conventional *P*<.05 level, the results are still notable given the small sample size (n=13). Nevertheless, model confidence does not reliably predict binding affinity at this stage.

The raw and calibrated probability distributions for each sequence are presented in Figures 10-12. The apparent improvement in the reliability of probability estimates does not reduce confidence. Calibration rescales overconfident outputs, so that the predicted values more closely reflect the true likelihood of correct classification, thereby producing interpretable probabilities that generalize more accurately to new experimental sequences.

**Figure 10.**
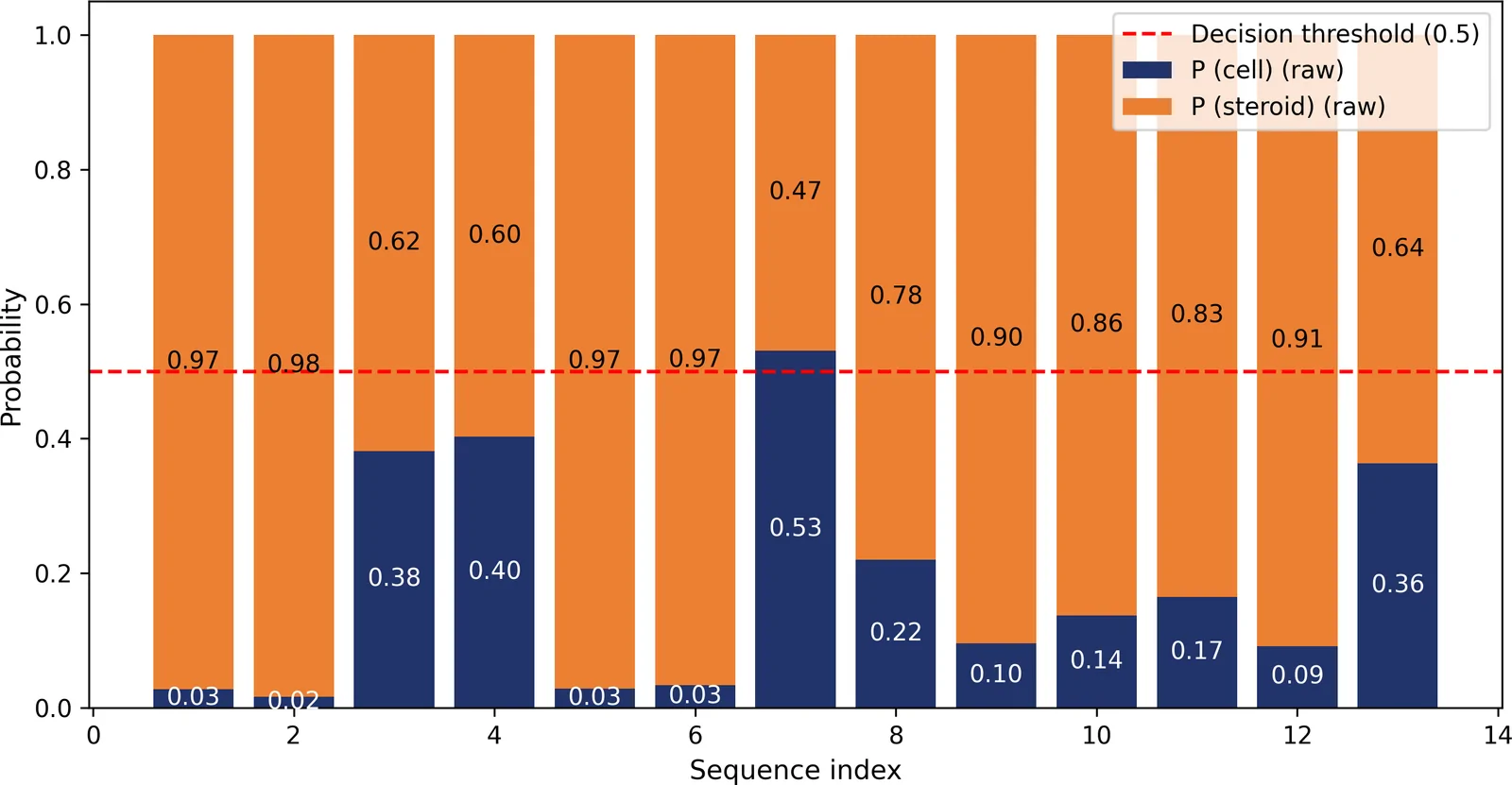
Distribution of raw computed probability values for all laboratory-generated experimental sequences.

**Figure 11.**
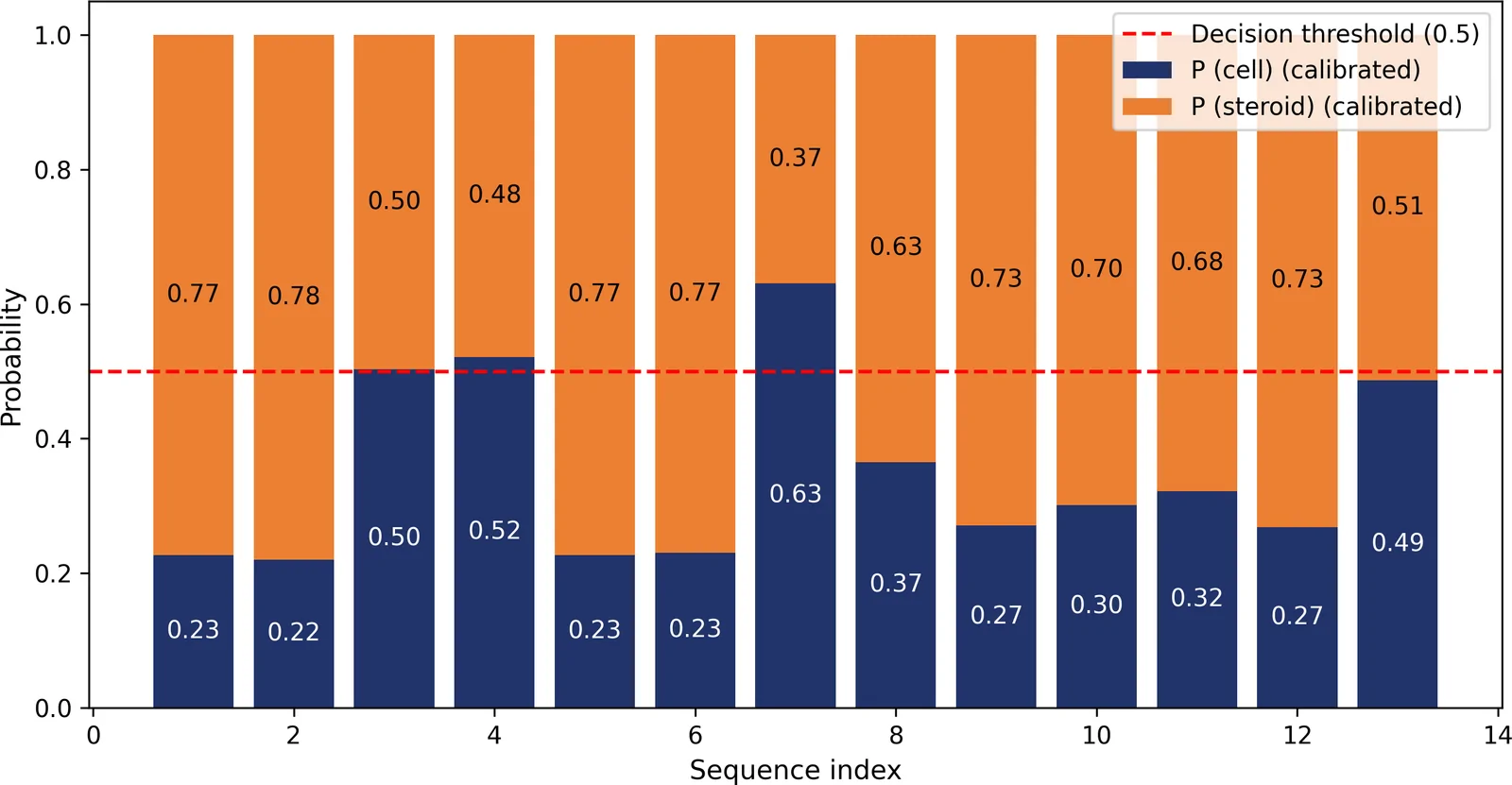
Distribution of calibrated computed probability values for all laboratory-generated experimental sequences.

**Figure 12.**
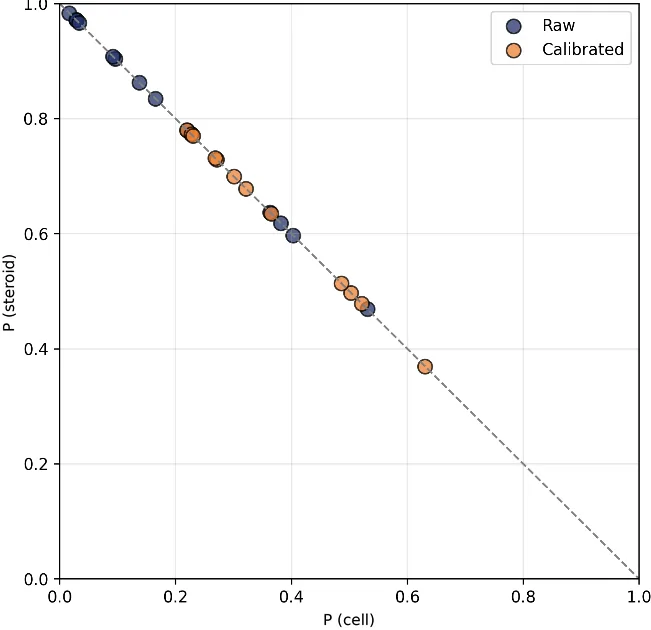
Scatter plot of raw vs computed probabilities sequence for all laboratory-generated sequences.

## Discussion

### Principal Findings

This preliminary study found that a sequence-only ensemble ML framework can accurately distinguish between cellular and steroidal DNA aptamers using TF-IDF motif representations. The calibrated XGBoost ensemble achieved strong classification performance on both the held-out test set and an independent set of experimentally derived steroid-binding aptamers, demonstrating the model’s ability to generalize beyond curated training datasets. These initial findings suggest that a purely sequence-based predictive framework can meaningfully complement wet-laboratory SELEX discovery by prescreening candidate sequences for binding potential. The ability to produce calibrated, interpretable probability estimates enables the selection of aptamer candidates for validation, potentially reducing experimental cycles and resource requirements. In addition, feature importance analysis revealed that the model potentially relied on recurring relevant sequence motifs and global sequence length for classification.

### Context Within the Literature

Prior approaches to aptamer discovery largely relied either on thermodynamic structure prediction (eg, NUPACK or MFold) or on training on aptamer-target pairs (eg, AptaBLE [12]). While these methods have significantly advanced the field, they are computationally expensive, scale poorly to large aptamer libraries, and are sensitive to inaccurate folding predictions. Moreover, models such as AptaBLE require retraining for each new target, limiting their applicability across the multiple categories commonly encountered in aptamer discovery. Hybrid methods, such as combining ML with genetic algorithms [18], have also been explored for aptamer design, but focus on generating aptamer candidates for a single predetermined target rather than classifying candidates across multiple target categories. By contrast, the sequence-only, TF-IDF framework presented here does not require structural modeling. It can be extended to multiple target classes, thereby providing a lightweight complement to the methods above.

### Practical Implications and Applications

In practice, a classifier of this kind could serve as an early triage step within an aptamer discovery pipeline: candidate sequences that emerge from initial SELEX rounds, in silico libraries, or public aptamer databases could be quickly scored for target-class compatibility before committing additional rounds of SELEX. In the evaluation case presented here, the model correctly classified all 13 laboratory-formulated SELEX-derived new ALDO aptamers as steroid-binding, achieving 100% recall on sequences confirmed to be genuinely novel to the training set (mean normalized Levenshtein distance 0.40, SD 0.07). As aptamer databases continue to grow, sequence-only classifiers of this kind could serve as a prescreening layer to quickly identify viable aptamer candidates for a target class, thereby reducing SELEX cycles and leading to faster, higher-throughput biosensor development.

### Limitations

Several limitations should be considered when interpreting the results of this study. The curated dataset was relatively small and imbalanced toward cell-binding aptamers, which may limit the diversity of sequence patterns learned by the model, despite the use of oversampling and class weighting. Additionally, although the model successfully generalized to 13 independently generated SELEX-derived steroid aptamers, further validation on larger, more diverse experimental datasets will be necessary to assess robustness across broader target classes. Finally, because the model selection was not benchmarked against alternative classifiers, the reported performance reflects the performance of the XGBoost ensemble specifically rather than a comparison with other algorithmic approaches, and it remains possible that other models could achieve comparable or improved performance on this task. Additionally, although feature importance analysis identified recurring motifs for classification, these only reflect statistical association within the dataset and model rather than actual structural roles in binding. Therefore, further investigation is needed to establish them as irrefutable determinants of target specificity.

### Future Work

Future work will extend this framework to multitarget classification. Regression on continuous K_d_ values, combined with structure-based features to capture higher-order sequence dependencies, will also be explored. Future work will also include systematic benchmarking against other classifiers to evaluate whether the observed performance is generalizable across various model architectures.

## Supplementary material

10.2196/92194 Multimedia Appendix 1 Predicted secondary structures and dissociation curves for 13 laboratory-generated steroid-binding aptamers.

## Abbreviations

ALDO: aldosterone
AptaBLE: Aptamer Binding Language
Caltech: California Institute of Technology
GC: guanine-cytosine
IDF: inverse document frequency
ML: machine learning
NUPACK: Nucleic Acid Package
PBS: phosphate-buffered saline
SELEX: systematic evolution of ligands by exponential enrichment
TF: term frequency
TF-IDF: term frequency–inverse document frequency
ThT: thioflavin T
XGBoost: extreme gradient boosting
