# Supplementary material for: Sequence-Driven Classification of Aptamers by Target Type: Algorithm Development and Validation
Source: JMIR Bioinform Biotechnol. 2026 Nov 13;7:e92194. doi: 10.2196/92194 (PMC13766662; doi:10.2196/92194)
Supplement: Supplementary file 1 [file bioinform-v7-e92194-s001.docx]

**Multimedia Appendix 1: Predicted secondary structures and dissociation curves for 13 lab-generated steroid-binding aptamers**

**Page**

**Predicted secondary structures from mFold and Nupack**

Figure S1. Predicted secondary structures of aldosterone aptamer 14	2

Figure S2. Predicted secondary structures of aldosterone aptamer 15	2

Figure S3. Predicted secondary structures of aldosterone aptamer 05	3

Figure S4. Predicted secondary structures of aldosterone aptamer 11	3

Figure S5. Predicted secondary structures of aldosterone aptamer 17	4

Figure S6. Predicted secondary structures of aldosterone aptamer 23	4

Figure S7. Predicted secondary structures of aldosterone aptamer 22	5

Figure S8. Predicted secondary structures of aldosterone aptamer 06	5

Figure S9. Predicted secondary structures of aldosterone aptamer 13	6

Figure S10. Predicted secondary structures of aldosterone aptamer 10	6

Figure S11. Predicted secondary structures of aldosterone aptamer 02	7

Figure S12. Predicted secondary structures of aldosterone aptamer 07	7

**Dissociation curves for ThT fluorescent assay**

Figure S13. ThT dissociation curve for aptamer 14	8

Figure S14. ThT dissociation curve for aptamer 15	9

Figure S15. ThT dissociation curve for aptamer 05	10

Figure S16. ThT dissociation curve for aptamer 11	11

Figure S17. ThT dissociation curve for aptamer 17	12

Figure S18. ThT dissociation curve for aptamer 23	13

Figure S19. ThT dissociation curve for aptamer 22	14

Figure S20. ThT dissociation curve for aptamer 06	15

Figure S21. ThT dissociation curve for aptamer 13	16

Figure S22. ThT dissociation curve for aptamer 10	17

Figure S23. ThT dissociation curve for aptamer 02	18

Figure S24. ThT dissociation curve for aptamer 07	19

Figure S25. ThT dissociation curve for aptamer 16	20

*Figure S1.* Predicted secondary structures of aldosterone aptamer 14. Generated images from mFold (left) and Nupack (right).

*Figure S2.* Predicted secondary structures of aldosterone aptamer 15. Generated images from mFold (left) and Nupack (right).

*Figure S3.* Predicted secondary structures of aldosterone aptamer 05. Generated images from mFold (left) and Nupack (right).

*Figure S4.* Predicted secondary structures of aldosterone aptamer 11. Generated images from mFold (left) and Nupack (right).

*Figure S5.* Predicted secondary structures of aldosterone aptamer 17. Generated images from mFold (left) and Nupack (right).

*Figure S6.* Predicted secondary structures of aldosterone aptamer 23. Generated images from mFold (left) and Nupack (right).

*Figure S7.* Predicted secondary structures of aldosterone aptamer 22. Generated images from mFold (left) and Nupack (right).

*Figure S8.* Predicted secondary structures of aldosterone aptamer 06. Generated images from mFold (left) and Nupack (right).

*Figure S9.* T Predicted secondary structures of aldosterone aptamer 13. Generated images from mFold (left) and Nupack (right).

*Figure S10.* Predicted secondary structures of aldosterone aptamer 10. Generated images from mFold (left) and Nupack (right).

*Figure S11.* Predicted secondary structures of aldosterone aptamer 02. Generated images from mFold (left) and Nupack (right).

*Figure S12.* Predicted secondary structures of aldosterone aptamer 07. Generated images from mFold (left) and Nupack (right).

*Figure S13.* Dissociation curve using 50μM ThT fluorescent dye and 5μM aptamer 14, in SELEX buffer with 2mM ${MgCl}_{2}$. Excitation and Emission for ThT are 425nm and 490nm, respectively. Technical triplicate samples pipetted into 384- well plate. $K_{d}$ results determined in Graph Pad Prism non-linear curve.

*Figure S14.* Dissociation curve using 50μM ThT fluorescent dye and 5μM aptamer 15, in SELEX buffer with 2mM ${MgCl}_{2}$. Excitation and Emission for ThT are 425nm and 490nm, respectively. Technical triplicate samples pipetted into 384- well plate. $K_{d}$ results determined in Graph Pad Prism non-linear curve.

*Figure S15.* Dissociation curve using 50μM ThT fluorescent dye and 5μM aptamer 05, in SELEX buffer with 2mM ${MgCl}_{2}$. Excitation and Emission for ThT are 425nm and 490nm, respectively. Technical triplicate samples pipetted into 384- well plate. $K_{d}$ results determined in Graph Pad Prism non-linear curve.

*Figure S16.* Dissociation curve using 50μM ThT fluorescent dye and 5μM aptamer 11, in SELEX buffer with 2mM ${MgCl}_{2}$. Excitation and Emission for ThT are 425nm and 490nm, respectively. Technical triplicate samples pipetted into 384- well plate. $K_{d}$ results determined in Graph Pad Prism non-linear curve.

*Figure S17.* Dissociation curve using 50μM ThT fluorescent dye and 5μM aptamer 17, in SELEX buffer with 2mM ${MgCl}_{2}$. Excitation and Emission for ThT are 425nm and 490nm, respectively. Technical triplicate samples pipetted into 384- well plate. $K_{d}$ results determined in Graph Pad Prism non-linear curve.

*Figure S18.* Dissociation curve using 50μM ThT fluorescent dye and 5μM aptamer 23, in SELEX buffer with 2mM ${MgCl}_{2}$. Excitation and Emission for ThT are 425nm and 490nm, respectively. Technical triplicate samples pipetted into 384- well plate. $K_{d}$ results determined in Graph Pad Prism non-linear curve.

*Figure S19.* Dissociation curve using 50μM ThT fluorescent dye and 5μM aptamer 22, in SELEX buffer with 2mM ${MgCl}_{2}$. Excitation and Emission for ThT are 425nm and 490nm, respectively. Technical triplicate samples pipetted into 384- well plate. $K_{d}$ results determined in Graph Pad Prism non-linear curve.

*Figure S20.* Dissociation curve using 50μM ThT fluorescent dye and 5μM aptamer 06, in SELEX buffer with 2mM ${MgCl}_{2}$. Excitation and Emission for ThT are 425nm and 490nm, respectively. Technical triplicate samples pipetted into 384- well plate. $K_{d}$ results determined in Graph Pad Prism non-linear curve.

*Figure S21.* Dissociation curve using 50μM ThT fluorescent dye and 5μM aptamer 13, in SELEX buffer with 2mM ${MgCl}_{2}$. Excitation and Emission for ThT are 425nm and 490nm, respectively. Technical triplicate samples pipetted into 384- well plate. $K_{d}$ results determined in Graph Pad Prism non-linear curve.

*Figure S22.* Dissociation curve using 50μM ThT fluorescent dye and 5μM aptamer 10, in SELEX buffer with 2mM ${MgCl}_{2}$. Excitation and Emission for ThT are 425nm and 490nm, respectively. Technical triplicate samples pipetted into 384- well plate. $K_{d}$ results determined in Graph Pad Prism non-linear curve.

*Figure S23.* Dissociation curve using 50μM ThT fluorescent dye and 5μM aptamer 02, in SELEX buffer with 2mM ${MgCl}_{2}$. Excitation and Emission for ThT are 425nm and 490nm, respectively. Technical triplicate samples pipetted into 384- well plate. $K_{d}$ results determined in Graph Pad Prism non-linear curve.

*Figure S24.* Dissociation curve using 50μM ThT fluorescent dye and 5μM aptamer 07, in SELEX buffer with 2mM ${MgCl}_{2}$. Excitation and Emission for ThT are 425nm and 490nm, respectively. Technical triplicate samples pipetted into 384- well plate. $K_{d}$ results determined in Graph Pad Prism non-linear curve.

*Figure S25.* Dissociation curve using 50μM ThT fluorescent dye and 5μM aptamer 16, in SELEX buffer with 2mM ${MgCl}_{2}$. Excitation and Emission for ThT are 425nm and 490nm, respectively. Technical triplicate samples pipetted into 384- well plate. $K_{d}$ results determined in Graph Pad Prism non-linear curve.
